# Association between sedentary behavior, physical activity, and neck pain, disability index, and cervical physiological function in university students: a cross-sectional study

**DOI:** 10.7717/peerj.20908

**Published:** 2026-03-09

**Authors:** Qiang Dong, Haojie Cheng, Hengjia Liu, Yawen Chang, Jiangyuan Li, Dongni Zhang

**Affiliations:** 1 Department of Physical Education, Pukyong National University, Busan, Republic of South Korea; 2 College of Physical Education, Henan Normal University, Henan, China; 3 Department of Physical Education, Zhengzhou Business University, Zhengzhou, China

**Keywords:** Sedentary behavior, Physical activity, Cervical health, Cervical physiological function

## Abstract

**Background:**

With changes in modern lifestyle, cervical health issues among college students have become increasingly prevalent.

**Objective:**

This study aimed to explore the associations between sedentary behavior (SB), physical activity (PA), and neck pain, disability index, and cervical physiological function in university students.

**Methods:**

This cross-sectional study enrolled 126 participants. Self-reported questionnaires were used to assess SB, PA, neck pain (VAS), and the Neck Disability Index (NDI). Objective measurements were taken to evaluate cervical range of motion (ROM), joint position sense (JPS), muscle strength (MS), and muscle endurance (ME). Pearson and Spearman correlations examined the associations between SB, PA, and cervical health indicators. Analysis of variance (ANOVA) and Kruskal-Wallis tests compared differences between groups with varying SB and PA levels.

**Results:**

Correlation analysis revealed significant positive correlations between SB and VAS , as well as JPS (extension JPS-E; right rotation JPS-RRot), and significant negative correlations with cervical ROM (extension ROM-E; left rotation ROM-LRot; right rotation ROM-RRot). Moreover, PA was negatively correlated with NDI and positively correlated with cervical flexor strength (CFS) and extensor strength (CES). Comparative analysis of different levels of SB and PA showed that the High-SB & Low-PA group had significantly worse outcomes in NDI, ROM-RRot, JPS-E, and CES.

**Conclusion:**

SB negatively affects cervical health in university students, whereas PA is associated with better neck function indicators. The combined effect of high SB and low PA exacerbates disability index, and further deteriorates cervical physiological function. This study provides empirical evidence for preventive and interventional strategies targeting cervical health issues in university students.

## Introduction

Recent years have witnessed a growing public health challenge regarding cervical health issues among university students (Arribas-Romano et al., 2024; Liu et al., 2025; Sezer et al., 2022), This has placed a considerable economic strain on healthcare systems (Kazeminasab et al., 2022). Epidemiological research has shown a notable rise in the prevalence of cervical disorders from adolescence to early adulthood (Jahre et al., 2021), with 48%–78% of university students reporting neck pain (Chan et al., 2020; Gao et al., 2023) and 35.6% experiencing moderate neck-related disability (Daher & Halperin, 2021). The high incidence of these diseases may be attributed to a variety of factors (Gao et al., 2023). Further analysis revealed that the behavioral patterns of university students exhibit significant risk characteristics. Recent epidemiological studies have described that university students sit for an average of 11.10 h daily (Moulin et al., 2021), with 40%–50% of individuals failing to meet basic physical activity standards (Martínez-Bello, Martínez-Rojas & Molina-García, 2017; Pengpid et al., 2015). This “high sedentary behavior, low physical activity” pattern can be primarily attributed to multiple factors such as prolonged desk work, dependence on electronic devices, and a lack of physical activity habits. Existing studies have established a close association between excessive sedentary behavior (SB), insufficient physical activity (PA), and adverse health indicators or outcomes, which has garnered extensive attention (Feter et al., 2024; Wshah et al., 2022; Xia et al., 2025). However, the mechanisms linking these behaviors to cervical health issues in university students remain to be systematically explored.

Existing studies have gradually focused on the association between SB, PA, and cervical health issues, along with the underlying pathogenic mechanisms, achieving notable progress. Specifically, concerning the relationship between SB, PA, and neck pain and disability index, several studies have demonstrated that a sedentary lifestyle not only significantly increases the risk of neck pain but also exacerbates the degree of disability index (García-Remeseiro et al., 2023; Meng et al., 2025). In contrast, the associations between PA, neck pain, and disability index are inconsistent. Some studies have suggested that physical activity effectively alleviates neck pain and disability index caused by prolonged sitting (Jones, Jadhakhan & Falla, 2024; Khan et al., 2024; Lee et al., 2025), while other studies have not identified significant associations (García-Remeseiro et al., 2023; Veseta et al., 2020), indicating that the effects of PA on cervical health may be specific to certain contexts.

Regarding the association between SB, PA, and musculoskeletal and physiological functions, epidemiological evidence suggests that reducing SB and increasing intermittent PA are beneficial for skeletal health (Ren et al., 2024). A meta-analysis further validated that higher PA levels and lower SB are significantly associated with improvements in skeletal muscle strength and muscle power (Ramsey et al., 2021). Additionally, earlier studies have pointed out that prolonged sitting and lack of PA reduce thoracic spine mobility (Heneghan et al., 2018) and lead to limitations in hip joint extension (Boukabache, Preece & Brookes, 2021). According to previous studies, chronic low back pain patients who remain sedentary exhibit significantly larger lumbar repositioning errors (Saiklang et al., 2024), whereas individuals who engage in regular exercise demonstrate superior joint position sense (Azevedo, Rodrigues & Seixas, 2021). Several longitudinal studies have indicated that exercise interventions can improve joint range of motion, muscle strength, and other functional outcomes (Akbaba et al., 2025; Reddy et al., 2021).

Regarding the potential pathogenic mechanisms by which SB and PA may influence cervical health, one commonly proposed pathway involves prolonged static postures. In particular, when engaging in academic tasks or using electronic devices, prolonged sitting is often accompanied by text-neck posture, This posture significantly increases the cervical spine’s forward flexion angle. Prolonged maintenance of this posture has been identified as a major risk factor for neck health deterioration. Biomechanically, an increase in the cervical spine’s forward flexion angle may elevate intervertebral joint pressure and alter spinal loading patterns (Barrett, McKinnon & Callaghan, 2020). It is important to note that while such postures are frequently implicated clinically and in biomechanical models, epidemiological evidence to date has not consistently established a direct causal link between specific text-neck postures and neck pain or disability. Nevertheless, from a mechanistic perspective, sustained text-neck postures is thought to contribute to increased muscular tension in the cervical stabilizers (Fercho et al., 2023), which may lead to soft tissue adaptation, stiffness, and reduced cervical mobility over time. These changes could, in turn, diminish cervical range of motion and impair proprioceptive function (Raunest, Sager & Burgener, 1996). From a metabolic perspective, SB significantly reduces skeletal muscle contraction activity, thereby inhibiting anabolic processes (Pesola et al., 2015). Clinical studies have corroborated that the combined effect of reduced muscle protein synthesis and increased breakdown triggers skeletal muscle loss in healthy individuals, with prolonged inactivity leading to a decline in muscle mass or muscle atrophy (Vainshtein & Sandri, 2020). In contrast, regular PA effectively improves muscle function and tendon adaptability (Grosset et al., 2014) while concurrently alleviating muscle stiffness (Kett, Milani & Sichting, 2021).

Despite advances made in current research on the relationship between SB, PA, and cervical health, several limitations remain: (1) Unidimensional indicators: Existing studies have primarily focused on the associations between SB, PA, and pain or disability index, with insufficient comprehensive investigation into cervical physiological function indicators. (2) Population specificity: Differences may exist between populations, and current studies lack a systematic analysis of cervical health issues specific to university students. (3) Heterogeneity of results: The majority of studies have treated SB and PA as independent or simple linear variables, overlooking the complexity of behavioral patterns and leading to conflicting results. (4) Limitations of statistical methods: Existing evidence has predominantly relied on simple correlation analyses, lacking categorical comparisons of different behavioral patterns among university students.

Based on these limitations, the present study was designed to fill the gaps in current research on university students and physiological function indicators. Through a cross-sectional design, this study aimed to explore the associations between SB, PA, and neck pain (VAS), neck disability index (NDI), and cervical physiological functions (range of motion, joint position sense, muscle strength, muscle endurance) among university students to expand our understanding of the potential mechanisms through which SB and PA impact cervical health in this population, thereby providing a basis for the development of scientific campus health intervention strategies.

## Materials and Methods

### Research study design

**Figure 1:**
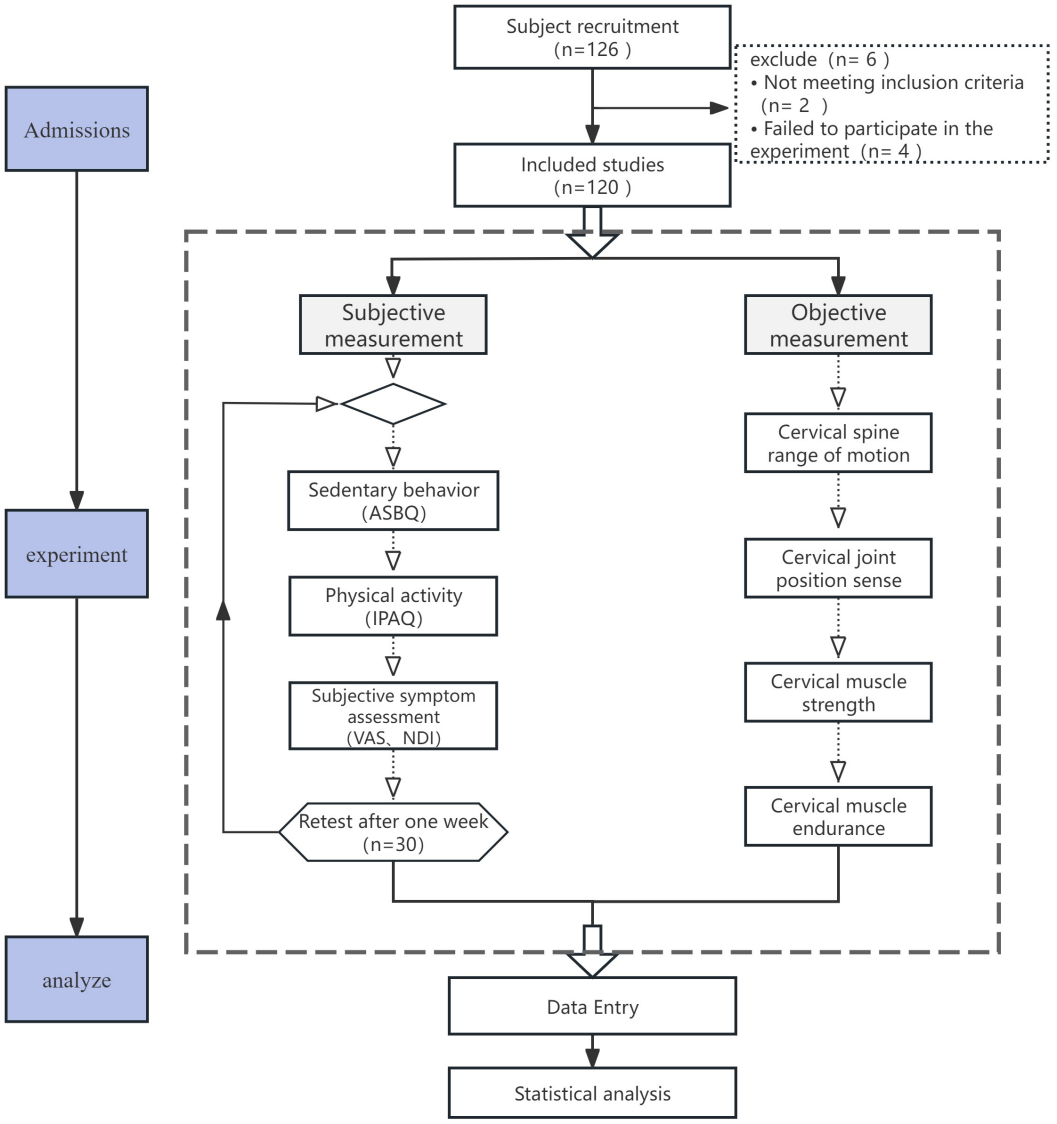
Research flowchart.

This study employed a cross-sectional survey design, with the primary procedure involving: (1) recruitment and screening of participants; (2) collection of data on SB, PA, and cervical health-related indicators through questionnaires and instrumental measurements; (3) correlation analyses of SB, PA, and cervical health indicators; (4) grouping participants based on different SB and PA levels for comparative analysis. This design effectively balanced the self-reported nature of behavioral data with objective physiological function measurements, ensuring the scientific rigor and practical relevance of the study’s findings. The specific research procedure is illustrated in Fig. 1. This study was conducted in full compliance with the ethical principles for human research as outlined in the Declaration of Helsinki and follows the STROBE reporting guidelines (Von Elm et al., 2007). The study has been approved by the Institutional Review Board of Pukyong National University (Number: PKNU202505006-UE001). Written informed consent was obtained from all participants before any data were collected.

### Participants

The sample size for this study was calculated using G*Power 3.1.9.4 software (University of Kiel, Kiel, Germany). The significance level (*α*) was set to 0.05, the effect size was set to 0.3, and the statistical power was set to 0.9. Based on these parameters, the minimum required sample size was 112 participants. Considering a 10% dropout rate, a total of 126 participants were recruited to ensure the reliability and validity of the data. We established the following inclusion criteria: age ≥18 years, BMI < 30, normal cognitive and functional abilities, and no prior history of cervical trauma or severe cervical diseases. We applied the following exclusion criteria: (1) cervical or thoracic spine fractures, dislocations, or a history of surgery; (2) history of musculoskeletal injuries; (3) history of osteoporosis, spinal infections, or fibromyalgia; (4) history of neurological disorders or recent treatment for cervical conditions. Ultimately, 120 eligible students were included in the study.

### Outcome measures

#### Subjective questionnaire assessment

Demographic data, including age, gender, academic level, height, weight, and BMI, were collected from participants. The Adult Sedentary Behavior Questionnaire (ASBQ) was used to assess sedentary behavior on weekdays and weekends across various domains. This questionnaire demonstrated satisfactory reliability and validity, with intraclass correlation coefficients (ICCs) for all items falling within an acceptable range of 0.66−0.74 (Chu et al., 2018). The International Physical Activity Questionnaire (IPAQ-short form) was used to assess the intensity and duration of various physical activities undertaken during the previous week. This widely used global assessment tool has demonstrated validity and reliability in college students, showing favorable psychometric properties, with an ICC range of 0.71−0.89 (Dinger, Behrens & Han, 2006). The Visual Analog Scale (VAS) was used to evaluate the intensity of neck pain, using a 10 cm horizontal line with two words describing extreme symptoms as reference points. Participants were instructed to mark a line on the vertical axis of the VAS to represent their neck pain intensity (Owen et al., 2019). The Neck Disability Index (NDI) questionnaire was adopted to evaluate neck functional impairment. The questionnaire comprises 10 items that evaluate pain and functional status. Each item is scored on a scale from 0 to 5, with the total score representing the overall disability level (Vernon, 2008).

#### Objective indicator measurement

Cervical range of motion (ROM) was assessed using a Cervical Range of Motion (C-ROM) device, which measured cervical flexion, extension, left rotation, and right rotation. Participants sat upright with their feet flat on the ground, and the CROM device was placed on their head. Prior to formal measurements, participants performed one trial in each direction to familiarize themselves with the measurement procedure and the range of motion. During testing, the difference in angle between the starting and ending positions in the natural posture of each direction was recorded as the ROM value. Each measurement was conducted three times, and the average value was then calculated (Fletcher & Bandy, 2008).

Cervical joint position sense was evaluated using the CROM device, which measured errors during cervical movements such as flexion, extension, and both left and right rotations. In brief, participants sat in an upright position in a chair with a backrest, ensuring that their hips and knees formed a 90-degree angle, while keeping their feet flat on the floor. A CROM device was then placed on the participant’s head. During the test, participants were instructed to close their eyes. First, cervical ROM was measured to determine the target head position. In the initial phase, the range of motion (ROM) in all directions of the neck was recorded. Based on this, the assessor gently passively positioned the subject’s head at the calculated midpoint (set to 50% of the maximum ROM) and held the position for 5 s. During this time, the subject was required to focus on memorizing the spatial orientation of the head. Then, the subject’s head was returned to the initial position, and the subject was instructed to actively move to the target position. Joint position sense was quantified by calculating the angular deviation (Alahmari et al., 2017). Each movement direction was tested three times, with the average taken as the final result. The entire test process is conducted by the same assessor, and verbal feedback is prohibited to ensure the objectivity of the results.

Cervical muscle strength was measured using a hand-held dynamometer (microFET2, Hoggan Health Industries) to assess isometric contraction strength of the cervical flexor and extensor muscle groups (Cools et al., 2010). This measurement method has demonstrated high reliability (ICC = 0.89−0.96). During the cervical flexor strength test, participants were positioned supine on the experimental table, and the hand-held dynamometer was placed under the chin. Participants were instructed to nod their head, pressing the chin downward against the dynamometer while maintaining resistance in the direction of cervical flexion (nodding). At the same time, the examiner palpated the sternocleidomastoid to monitor and provide feedback, preventing the activation of superficial flexors (Suvarnnato et al., 2019). For the cervical extensor strength measurement, participants were positioned prone, with their shoulders resting at the edge of the experimental table and their head in an anti-gravity posture. The dynamometer was placed at the center of the participant’s occiput. The examiner applied force in the direction of cervical flexion while the participant maintained a neutral neck position. A 1-minute rest was allocated between each measurement.After three measurements, the average value was calculated and used for analysis. The force values (N) for each muscle were normalized to body weight (kg).

Cervical muscle endurance was tested using a stopwatch. During the cervical flexor endurance test, participants assumed a flexed position while lying on the experimental table. The examiner placed their right hand under the participant’s occiput, slightly flexing the neck. Afterward, participants were instructed to gently bend the upper neck and lift their head off the examiner’s hand while maintaining the flexion. The test was terminated when the participant’s head touched the examiner’s left hand or could not maintain their head off the examiner’s hand. The duration of time was measured in seconds (Harris et al., 2005). For the cervical extensor endurance test, participants were positioned prone on the experimental table, with their arms by their sides and their head at the edge of the table. Thereafter, a strap was positioned at the T6 level to stabilize the upper thoracic spine. During the experiment, the subject wore a head-mounted device equipped with an inclinometer. Males were instructed to suspend a 4 kg weight, and females a 2 kg weight (initially positioned just below the ground). The subject was required to maintain a neutral cervical posture in the horizontal plane. The test was terminated when the suspended weight made contact with the ground or when the cervical angle deviated more than 5° from the horizontal position. The duration of the test was recorded in seconds (Edmondston et al., 2008).

### Statistical analysis

Statistical analysis was conducted using SPSS version 27.0, while graphical representations were generated using GraphPad Prism 10.5. The study was divided into three primary stages: (1) First, descriptive statistics were applied to summarize the fundamental characteristics of the variables. Continuous variables were reported as means, standard deviations, maximum values, and minimum values, while categorical variables were presented as absolute frequencies (n) and relative frequencies (%). (2) The Kolmogorov–Smirnov test was used to assess the normality of the data, and Pearson or Spearman correlation analyses were conducted to explore the strength and direction of the relationships between SB, PA, and neck pain, disability index, and cervical physiological function among university students. (3) Based on the classification of participants according to their SB and PA levels, differences between groups were compared. Following the classification method proposed by Pinto, participants with an average daily sedentary time of ≥9.5 h were categorized as having high SB, and those engaging in less than 150 min of moderate-to-vigorous PA per week or less than 75 min of vigorous PA per week were classified as low PA. This led to four distinct categories, namely high SB with high PA, high SB with low PA, low SB with high PA, and low SB with low PA (Pinto et al., 2023). To mitigate the impact of small sample sizes on the results, the low sedentary behavior and low physical activity group (*n* = 6) was excluded. The remaining three groups were compared using one-way analysis of variance (ANOVA) or non-parametric tests (Kruskal–Wallis) to evaluate differences in neck pain, disability index, and cervical physiological function. The significance threshold for all statistical analyses was set at 5% (*p* < 0.05).

## Results

### Demographic information and descriptive statistics

A total of 126 university students were recruited for the study. However, two participants did not meet the inclusion criteria, and four participants did not complete the experiment in its entirety. Therefore, data from 120 participants were ultimately included in the analysis, consisting of 62 males (51.7%) and 58 females (48.3%). The average age of all participants was 26.32 ± 4.73 years (range: 18–37 years), with the majority aged between 20 and 30 years. The average height, weight, and body mass index (BMI) were 170.82 ± 9.00 cm (range: 155–200 cm), 67.12 ± 13.56 kg (range: 45–115 kg), and 22.76 ± 2.77 (range: 17.53–29.20), respectively, all of which met the inclusion criteria for the study.

Descriptive statistics for the participants revealed the following: the average daily sedentary time was 11.94 ± 3.13 h. Meanwhile, the average physical activity level was 2,614.93 ± 2,077.59 MET-min/week, with considerable interindividual variability. Regarding neck pain and disability index, the average neck pain score (VAS) was 2.37 ± 1.69, and the average Neck Disability Index (NDI) score was 6.15 ± 4.05. Concerning cervical physiological function, the range of motion (ROM) was 58.53 ± 9.55° for cervical flexion (ROM-F), 71.90 ± 11.81° for extension (ROM-E), 71.47 ± 8.21° for left rotation (ROM-LRot), and 66.83 ± 8.58° for right rotation (ROM-RRot). The joint position sense (JPS) errors were 3.12 ± 2.16° for cervical flexion (JPS-F), 3.59 ± 2.34° for extension (JPS-E), 4.01 ± 2.33° for left rotation (JPS-LRot), and 4.14 ± 2.78° for right rotation (JPS-RRot). At the same time, the cervical flexor strength (CFS) was 0.41 ± 0.11 N/kg, while the extensor strength (CES) was 0.70 ± 0.16 N/kg. The cervical flexor endurance (CFE) was 76.73 ± 45.33 s, and the extensor endurance (CEE) was 155.90 ± 66.41 s. These basic demographic and health parameters offer essential information regarding the participants’ overall health status and cervical health, laying a foundation for subsequent analysis. Detailed results are presented in Table 1.

**Table 1:** Participant characteristics and descriptive statistics (*n* = 120).

**Variable**	**Mean ± SD**	**Minimum-Maximum**
Age (years)	26.32 ± 4.73	18-37
Gender, n (%)	Male 62 (51.70%) Female 58 (48.30%)	
Educational level, n (%)	Undergraduate 28 (23.30%) Master’s Degree 42 (35.00%) Doctoral Degree 50 (41.70%)	
Height (cm)	170.82 ± 9.00	155–200
Weight (kg)	67.12 ± 13.56	45–115
BMI (kg/m^2^)	22.76 ± 2.77	17.53–29.20
SB (h/day)	11.94 ± 3.13	5.33–18.86
PA (MET-min/week)	2,614.93 ± 2,077.59	297.00–9,972.00
VAS (score)	2.37 ± 1.69	0–7.00
NDI (score)	6.15 ± 4.05	0–18.00
ROM-F (°)	58.53 ± 9.55	32.00–78.67
ROM-E (°)	71.90 ± 11.80	42.67–99.33
ROM-LRot (°)	71.47 ± 8.21	49.67–96.00
ROM-RRot (°)	66.83 ± 8.58	48.00–88.67
JPS-F (°)	3.12 ± 2.16	0–10
JPS-E (°)	3.59 ± 2.34	0–10.67
JPS-LRot (°)	4.01 ± 2.33	0.33–11.67
JPS-RRot (°)	4.14 ± 2.78	0.66–13.67
CFS (N/kg)	0.41 ± 0.11	0.21–0.72
CES (N/kg)	0.70 ± 0.16	0.40–1.08
CFE (s)	76.73 ± 45.33	13–244
CEE (s)	155.90 ± 66.41	40–365

**Notes.**

SB Sedentary behavior PA Physical activity VAS Visual analogue scale NDI Neck disability index ROM-F Range of motion-Flexion ROM-E Range of motion-Extension ROM-LRot Range of Motion -Left Rotation ROM-RRot Range of Motion-Right Rotation JPS-F Joint position sense-Flexion JPS-E Joint position sense-Extension JPS-LRot Joint position sense-Left Rotation JPS-RRot Joint position sense-Right Rotation CFS Cervical flexor strength CES Cervical extensor strength CFE Cervical flexor endurance CEE Cervical extensor endurance

### Correlation analysis between SB, PA, and cervical health indicators

Significant correlations were identified between SB and multiple cervical health indicators. Specifically, SB was significantly positively correlated with neck pain scores (VAS) (*r* = 0.198, *p* = 0.030), indicating that longer sedentary durations were associated with higher neck pain severity. Additionally, cervical range of motion (ROM) in extension (ROM-E, *r* =  − 0.191, *p* = 0.037), left rotation (ROM-LRot, *r* =  − 0.238, *p* = 0.009), and right rotation (ROM-RRot, *r* =  − 0.203, *p* = 0.026) were significantly negatively correlated with SB, suggesting that prolonged sitting may limit cervical mobility. SB was also significantly correlated with joint position sense (JPS) errors in extension (JPS-E, *r* = 0.270, *p* = 0.003) and right rotation (JPS-RRot, *r* = 0.189, *p* = 0.039).

Regarding the relationship between PA and cervical health indicators, the analysis implied that PA was significantly negatively correlated with neck disability index (NDI) (*r* =  − 0.282, *p* = 0.002), suggesting that higher levels of PA may assist in alleviating neck disability index. Regarding cervical physiological function, PA was only significantly correlated with muscle strength. Specifically, cervical flexor strength (CFS) (*r* = 0.260, *p* = 0.004) and cervical extensor strength (CES) were positively correlated with PA (*r* = 0.260, *p* = 0.022), indicating that higher levels of PA may contribute to improved cervical muscle strength.

Overall, SB was negatively associated with neck pain, cervical range of motion, and joint position sense, while PA exerted positive effects on neck disability index and cervical muscle strength. Detailed results are presented in Table 2.

**Table 2:** Correlation between SB, PA, and cervical health indicators.

**Variable**	**SB**					**PA**				
	Analysis type	r	*p*	95% CI		Analysis type	r	*p*	95% CI	
				Lower	Upper				Lower	Upper
VAS	Spearman	0.198	0.030^*^	0.015	0.369	Spearman	−0.171	0.061	−0.345	0.013
NDI	Spearman	0.084	0.362	−0.102	0.264	Spearman	−0.282	0.002^**^	−0.443	−0.102
ROM-F	Pearson	−0.053	0.568	−0.230	0.128	Spearman	0.102	0.267	−0.084	0.281
ROM-E	Pearson	−0.191	0.037^*^	−0.358	−0.012	Spearman	0.049	0.595	−0.137	0.231
ROM-LRot	Pearson	−0.238	0.009^**^	−0.400	−0.061	Spearman	0.043	0.645	−0.143	0.225
ROM-RRot	Pearson	−0.203	0.026^*^	−0.369	−0.024	Spearman	0.111	0.229	−0.075	0.289
JPS-F	Spearman	0.034	0.714	−0.152	0.217	Spearman	−0.036	0.693	−0.219	0.149
JPS-E	Spearman	0.270	0.003^**^	0.090	0.433	Spearman	−0.110	0.231	−0.289	0.076
JPS-LRot	Spearman	0.051	0.583	−0.135	0.233	Spearman	−0.031	0.740	−0.214	0.155
JPS-RRot	Spearman	0.189	0.039^*^	0.005	0.361	Spearman	−0.019	0.834	−0.203	0.166
CFS	Spearman	−0.094	0.309	−0.214	0.154	Spearman	0.260	0.004^**^	0.017	0.372
CES	Pearson	0.005	0.953	−0.174	0.184	Spearman	0.209	0.022^*^	0.026	0.379
CFE	Spearman	−0.124	0.177	−0.302	0.062	Spearman	0.130	0.156	−0.055	0.307
CEE	Spearman	0.132	0.151	−0.054	0.309	Spearman	0.061	0.505	−0.124	0.243

**Notes.**

SB Sedentary behavior PA Physical activity VAS Visual analogue scale NDI Neck disability index ROM-F Range of motion-Flexion ROM-E Range of motion-Extension ROM-LRot Range of Motion -Left Rotation ROM-RRot Range of Motion-Right Rotation JPS-F Joint position sense-Flexion JPS-E Joint position sense-Extension JPS-LRot Joint position sense-Left Rotation JPS-RRot Joint position sense-Right Rotation CFS Cervical flexor strength CES Cervical extensor strength CFE Cervical flexor endurance CEE Cervical extensor endurance r Correlation Coefficient p statistical significance CI Confidence Interval

* *p* < 0.05.

** *p* < 0.01.

### Analysis of differences among groups with different SB and PA levels

**Figure 2:**
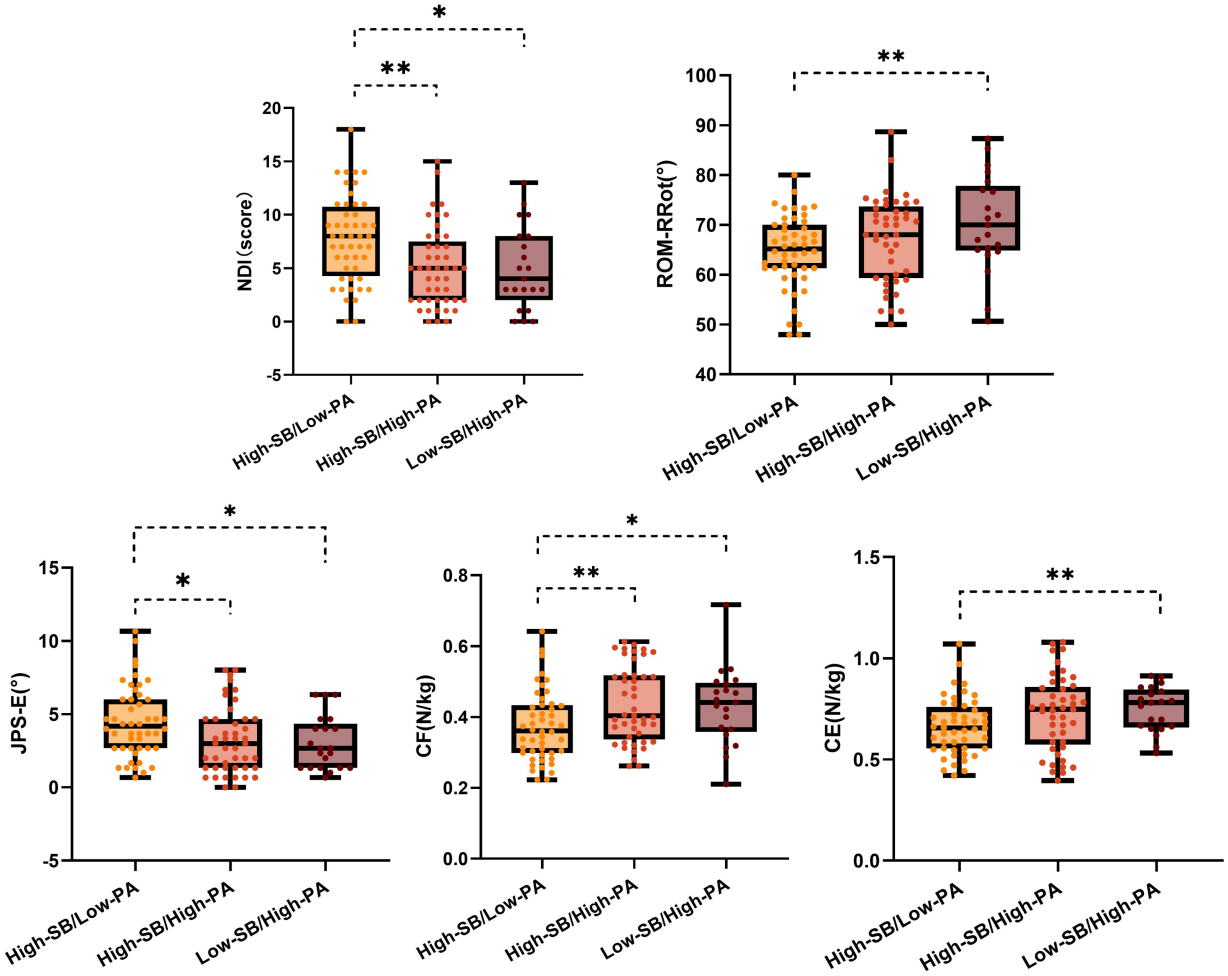
Differences among groups with different SB and PA levels. The *x*-axis of the box plot represents the grouping of different behavioral patterns, while the *y*-axis indicates the measured variable. Each box displays the interquartile range of the data, with the line inside the box representing the median. The whiskers extending from the box indicate the maximum and minimum values of the data, and individual points represent the values of each participant. Asterisks denote statistical significance, with the number of asterisks reflecting the degree of difference between the groups.

Differences in neck pain, neck disability index, and cervical physiological function indicators among groups with different levels of SB and PA (High-SB&Low-PA, High-SB&High-PA, and Low-SB&High-PA) were compared using the Kruskal–Wallis test and ANOVA. Figure 2 presents box plots displaying the distribution of cervical health indicators between the three groups, with the *x*-axis representing group categories and the *y*-axis indicating the measured values for each indicator.

Significant differences were noted in several cervical health indicators among groups with different levels of SB and PA. Specifically, in terms of neck disability index, significant group differences were observed in NDI scores (*H* = 12.11, *p* = 0.002). Specifically, the High-SB&Low-PA group (median = 8.00, IQR = 10.75–4.25) had significantly higher NDI scores compared to the High-SB&High-PA group (median = 7.50, IQR = 7.50–2.00, *p* = 0.001) and the Low-SB&High-PA group (median = 4.00, IQR = 8.00–2.00, *p* = 0.013). However, the VAS pain scores were comparable among the groups (*H* = 4.52, *p* = 0.104).

Analysis of cervical physiological function indicators revealed significant group differences in ROM-RRot (*F* = 3.63, *p* = 0.030). The High-SB&Low-PA group (64.63° ± 7.42) had significantly lower ROM-RRot compared to the Low-SB&High-PA group (70.41° ± 9.66, *p* = 0.009), while no significant differences were observed between other groups. Moreover, significant group differences were detected in JPS-E scores (*H* = 8.45, *p* = 0.015), with the High-SB&Low-PA group (median = 4.17, IQR = 6.00−2.67) showing significantly higher scores compared to both the High-SB&High-PA group (median = 3.00, IQR = 4.67−1.33, *p* = 0.012) and the Low-SB&High-PA group (median = 2.67, IQR = 4.33–1.33, *p* = 0.020).

Likewise, significant group differences were noted in cervical flexor strength (CFS) (*H* = 8.84, *p* = 0.012), with the High-SB&Low-PA group (median = 0.36, IQR = 0.43−0.30) displaying significantly lower strength than both the High-SB&High-PA group (median = 0.40, IQR = 0.52−0.34, *p* = 0.008) and the Low-SB&High-PA group (median = 2.67, IQR = 4.33−1.33, *p* = 0.020). Similarly, significant differences were observed in cervical extensor strength (CES) (*H* = 7.06, *p* = 0.029), with the High-SB&Low-PA group (median = 0.66, IQR = 0.76−0.55) exhibiting significantly lower strength than the Low-SB&High-PA group (median = 0.75, IQR = 0.86−0.57, *p* = 0.008). However, VAS, cervical range of motion (ROM-F, ROM-E, ROM-LRot), joint position sense (JPS-F, JPS-LRot, JPS-RRot) errors, and muscle endurance (CFE, CEE) were similar across groups (*p* > 0.05).

These results collectively suggest that high SB combined with low PA levels is significantly associated with poorer cervical function, with specific differences detailed in Table 3.

**Table 3:** Analysis of differences among groups with different SB and PA levels.

**Variables** **(*n* = 114)**	**Group**			**H/F**	***p***	***Post-hoc* tests**
	**High-SB&Low-PA** **(*n* = 48)**	**High-SB&High-PA** **(*n* = 45)**	**Low-SB&High-PA** **(*n* = 21)**			
VAS	3.00 (4.00–1.25)	2.00 (3.00–1.00)	1.00 (3.25–0.00)	4.52	0.104	–
NDI	8.00 (10.75–4.25)	5.00 (7.50–2.00)	4.00 (8.00–2.00)	12.11	0.002^**^	*a* > *b* (*p* = 0.001) *a* > *c* (*p* = 0.013)
ROM-F	58.35 ± 9.33	59.24 ± 10.21	58.60 ± 8.56	0.11	0.9	–
ROM-E	68.65 ± 12.33	73.99 ± 11.61	73.68 ± 9.68	2.83	0.063	–
ROM-LRot	70.94 ± 9.05	70.96 ± 7.25	73.32 ± 7.84	0.72	0.489	–
ROM-RRot	64.63 ± 7.42	67.15 ± 8.60	70.41 ± 9.66	3.63	0.030^*^	*a* < *c* (*p* = 0.009)
JPS-F	2.50 (4.00–1.75)	2.67 (4.00–1.67)	2.67 (4.5–1.33)	0.38	0.828	–
JPS-E	4.17 (6.00–2.67)	3.00 (4.67–1.33)	2.67 (4.33–1.33)	8.45	0.015^*^	*a* > *b* (*p* = 0.012) *a* > *c* (*p* = 0.020)
JPS-LRot	4.33 (6.00–2.42)	3.33 (5.33–2.00)	2.67 (5.00–2.00)	4.54	0.103	–
JPS-RRot	4.33 (6.00–2.33)	3.33 (5.33–2.00)	3.00 (5.67–2.00)	1.51	0.470	–
CFS	0.36 (0.43–0.30)	0.40 (0.52–0.34)	0.44 (0.50–0.36)	8.84	0.012^*^	*a* < *b* (*p* = 0.008) *a* < *c* (*p* = 0.025)
CES	0.66 (0.76–0.55)	0.75 (0.86–0.57)	0.78 (0.85–0.66)	7.06	0.029^*^	*a* < *c* (*p* = 0.008)
CFE	58.50 (86.75–38.00)	75.00 (101.00–52.00)	87.00 (126.00–47.50)	4.83	0.089	–
CEE	144.00 (200.50–109.25)	153.00 (205.00–109.50)	145.00 (196.00–117.00)	0.18	0.916	–

**Notes.**

High-SB/Low-PA High sedentary&low physical activity High-SB/High-PA High sedentary&high physical activity Low-SB/High-PA Low sedentary&high physical activity VAS Visual analogue scale NDI Neck disability index ROM-F Range of motion-Flexion ROM-E Range of motion-Extension ROM-LRot Range of Motion -Left Rotation ROM-RRot Range of Motion-Right Rotation JPS-F Joint position sense-Flexion JPS-E Joint position sense-Extension JPS-LRot Joint position sense-Left Rotation JPS-RRot Joint position sense-Right Rotation CFS Cervical flexor strength CES Cervical extensor strength CFE Cervical flexor endurance CEE Cervical extensor endurance

a High-SB/Low-PA.

b High-SB/High-PA.

c Low-SB/High-PA.

* *p* < 0.05.

** *p* < 0.01.

## Discussion

This study systematically explored the relationship between SB, PA levels, and cervical health indicators in university students. As anticipated, the results indicate that SB is significantly correlated with several cervical health indicators, particularly negatively affecting neck pain, cervical range of motion, and joint position sense. In contrast, PA is significantly associated with better neck function indicators. Group comparisons based on behavioral patterns revealed that the high sedentary behavior and low physical activity group generally performed worse on all cervical health indicators, suggesting that this behavioral pattern not only significantly exacerbates neck pain and disability index but also further impairs cervical physiological function. To the best of our knowledge, this is the first cross-sectional survey conducted among university students to explore the association between sedentary behavior, physical activity, and neck health indicators. Notably, this research not only elucidates the complex association between behavioral patterns and cervical health but also provides key empirical evidence for cervical health interventions targeted at university students.

The results of this study align with previous research findings. Extended periods of static postures have been recognized as a major health risk (Black, DesRoches & Arsenault, 2012), and earlier studies have established a link between SB and disorders of the neck and upper limbs, with potential underlying mechanisms involving various pathophysiological changes (Guduru et al., 2022). Moreover, research has highlighted the strong connection between prolonged sitting and the development of upper cross syndrome, as well as changes in upper limb function (Khawar et al., 2022). Notably, several studies have shown a significant association between SB and neck pain in adults (Mazaheri-Tehrani et al., 2023; Meng et al., 2025). Although research on the impact of SB on cervical physiological function is relatively limited, our findings are still supported by theoretical evidence: previous studies have evinced that static postures significantly restrict the range of motion of joints, and sitting posture negatively affects proprioceptive accuracy in cervical extension (Diong et al., 2019; Kim, Shin & Kim, 2021).

Concerning the association between PA and cervical health indicators, this study supports the association between physical activity and neck health indicators. Previous research has signaled that PA levels in university students are negatively correlated with neck disability index scores (Tao et al., 2024), while another longitudinal study concluded that exercise interventions can improve neck disability index (Elabd & Elabd, 2022). These findings can be ascribed to adaptive changes in the musculoskeletal system. For instance, earlier studies have reported that PA significantly impacts overall muscle strength and bone density (Nordstrom, Nordstrom & Lorentzon, 1997). At the same time, a recent study demonstrated that physical exercise enhances cervical muscle strength and alleviates pain (Heng et al., 2022). It is worthwhile emphasizing that while several studies have corroborated the beneficial effects of PA on neck disability index and muscle strength, some have reported no significant correlation with muscle endurance (Can & Tuna, 2022), which is in agreement with the results of this study.

This study reveals the differential impact of various SB and PA combination patterns on cervical health, with the high SB and low PA group displaying poorer performance on multiple cervical health indicators. This finding is corroborated by existing research evidence. Specifically, previous research has demonstrated that the combination of prolonged sedentary behavior and insufficient physical activity has a compounded detrimental effect on overall health (Alosaimi et al., 2023; Farrahi et al., 2022). In the context of cervical health, a prospective cohort study indicated that increasing step counts and reducing SB are key protective factors for preventing neck pain (Sitthipornvorakul, Janwantanakul & Lohsoonthorn, 2015). This protective mechanism may involve multiple factors. An earlier study found that individuals with more than 7 h of daily SB and less than 150 min of weekly PA had significantly reduced thoracic range of motion (Heneghan et al., 2018), while those with higher PA combined with lower SB exhibited improved skeletal muscle strength and muscle power (Ramsey et al., 2021). More importantly, a study recruiting student populations found that prolonged sitting and low PA levels lead to trunk muscle imbalance (Marijančić et al., 2023), which may provide a potential explanatory mechanism for the cervical function abnormalities observed in this study. These observations conjointly suggest that the interactions between SB and PA may be key factors influencing cervical health.

The findings of this study indicate that both the independent effects of SB and PA, as well as their interaction, contribute to varying degrees of cervical health deterioration. This can be attributed to biomechanical changes and physiological mechanisms. Studies have inferred that prolonged static sitting, particularly during head-down posture associated with learning, alters cervical joint load due to cervical flexion. Compared to a neutral posture, flexion increases the compressive force on the entire cervical spine by 1.6-fold (Barrett, McKinnon & Callaghan, 2020). This mechanical change can lead to neck muscle stiffness and a reduction in elasticity (Nitta et al., 2025), resulting in a vicious cycle of “mechanical abnormality-muscular change-limited joint mobility”. Regular physical activity, compared to sedentary behavior, is more beneficial for improving cervical microcirculation (Chia et al., 2017), offering a novel perspective for understanding the protective mechanism of PA. Although the exact mechanism by which SB and PA affect proprioception remains to be fully elucidated, previous studies have identified key mechanisms. For example, training can induce morphological adaptations in muscle spindles, including metabolic changes in intrafusal muscle fibers, a shortening in stretch reflex latency, and an increase in amplitude (Hutton & Atwater, 1992). Subsequent studies proposed the “force-control-sensation” hypothesis, proposing that strength training enhances motor control abilities, which in turn improves joint proprioception (Petrella, Lattanzio & Nelson, 1997). These findings collectively build a theoretical framework encompassing “peripheral receptor adaptation-central nervous regulation-overall functional improvement”, which not only provides scientific evidence for interpreting the findings of this study but highlights strategies for targeted interventions, including reducing SB to interrupt the mechanical vicious cycle, promoting neuromuscular remodeling through PA, and adopting a synergistic intervention approach to maintain cervical health.

Nevertheless, several limitations of this study merit acknowledgment. To begin, the exclusive recruitment of participants from a single Korean university may restrict the generalizability of findings due to restricted sample diversity and size. Furthermore, the cross-sectional methodology precludes causal inference regarding variable relationships. Consequently, future longitudinal designs tracking temporal dynamics are imperative to elucidate the chronic effects of SB and PA on cervical spine health. Thirdly, despite efforts to minimize measurement errors, the self-reported nature of SB/PA assessments may still introduce inherent biases. Based on these limitations, future research are recommended to address the following areas: (1) employing a multi-center design to increase sample diversity; (2) combining equipment-based measurement of behavior patterns to improve data accuracy; (3) conducting longitudinal studies to further validate the causal relationship between SB, PA, and cervical health indicators.

This study offers valuable insights for cervical health interventions. The results suggest that a synergistic intervention strategy aimed at reducing SB and concurrently increasing PA may effectively enhance cervical health among university students, including alleviating pain, improving functional impairment, and enhancing cervical physiological function. Universities are recommended to translate these findings into practical applications: (1) Breaking the sedentary pattern: Encourage regular physical activity or exercise to interrupt the sedentary pattern, thereby reducing the negative impact of static load on the neck; (2) optimizing workstation layout: Promote the use of adjustable-height standing desks, and encourage alternating between sitting and standing during work and study to reduce sedentary time; (3) scientifically managing screen usage: Standardize posture during electronic device use and control usage time to avoid abnormal pressure on the cervical spine caused by forward head posture; (4) creating a healthy learning environment: Provide students with ergonomically designed chairs and assistive tools such as reading stands and document holders to maintain proper reading and writing postures, thereby reducing neck strain caused by poor posture. These measures not only help improve cervical health among students but also exert a positive impact on their learning efficiency and quality of life. Furthermore, the findings hold considerable implications for health promotion among other sedentary populations (*e.g.*, office workers) and provide scientific evidence for public health policy development. Future research should further validate the effects of these interventions in different populations and assess their long-term benefits.

## Conclusion

This study systematically elucidated the associations between SB, PA, and neck pain, disability index, and cervical physiological function among university students. The results indicate that SB is significantly correlated with neck pain and cervical physiological function indicators, PA is significantly associated with better neck function indicators. The combination of high SB and low PA exerts the most significant negative impact on cervical health. The findings of this study provide guidance for health interventions in campus and workplace settings, suggesting that reducing SB and increasing PA may aid in improving cervical health in university students and other highly sedentary populations. Moreover, the results offer scientific evidence for developing targeted health intervention strategies with broad applicability. Future research should focus on establishing causal relationships through longitudinal tracking, optimizing exercise intervention programs, and developing personalized health management strategies. Finally, these findings hold significant public health significance for improving cervical health and quality of life among sedentary populations.

##  Supplemental Information

10.7717/peerj.20908/supp-1 Supplemental Information 1 Raw Data

10.7717/peerj.20908/supp-2 Supplemental Information 2 Codebook

10.7717/peerj.20908/supp-3 Supplemental Information 3 STROBE Checklist

## Additional Information and Declarations

### Data Availability

The following information was supplied regarding data availability: The raw measurements are available in the Supplemental File.
